# Volatile Organic Compounds and Beyond: Chemical Diversity and Biological Activities of *Dittrichia viscosa*

**DOI:** 10.3390/molecules31142474

**Published:** 2026-07-15

**Authors:** Martina Ghidoli, Emanuela Talarico, Diana-Maria Mircea, Eleonora Greco, Leonardo Bruno, Salvatore Roberto Pilu, Fabrizio Araniti

**Affiliations:** 1 Department of Agricultural and Environmental Sciences—Production, Landscape and Agroenergy, University of Milan, Via Celoria 2, 20133 Milan, Italy; martina.ghidoli@unimi.it (M.G.); salvatore.pilu@unimi.it (S.R.P.); 2 Department of Biology, Ecology and Earth Sciences (DiBEST), University of Calabria, 87036 Rende, Italy; emanuela.talarico@unical.it (E.T.); eleonora.greco@unical.it (E.G.); leonardo.bruno@unical.it (L.B.); 3 Mediterranean Agroforestry Institute (IAM), Universitat Politècnica de València, Camino de Vera s/n, 46022 Valencia, Spain; dmircea@doctor.upv.es; 4 Department of Horticulture and Landscape, University of Agricultural Sciences and Veterinary Medicine Cluj-Napoca, 3–5 Manastur Street, 400372 Cluj-Napoca, Romania

**Keywords:** *Dittrichia viscosa*, volatile organic compounds, sesquiterpenoids, glandular trichomes, biological activities

## Abstract

*Dittrichia viscosa* (L.) Greuter is a Mediterranean species widely recognized for its remarkable ecological plasticity and its rich repertoire of secondary metabolites, among which volatile organic compounds (VOCs) play a central role. This review provides an integrative synthesis of current knowledge on *D. viscosa*, with a primary focus on its VOCs, including their extraction techniques, chemotypic variability, and biological activities, while contextualizing these compounds within the species’ broader phytochemical and ecological framework. Available evidence indicates that VOC profiles are dominated by terpenoids, particularly oxygenated sesquiterpenes and monoterpenes. The composition of volatile fractions is strongly influenced by extraction methodology, plant organ, developmental stage, and geographic origin, resulting in pronounced chemotypic variability across Mediterranean populations. Beyond their direct bioactivity, VOCs are considered in their ecological context, particularly in relation to glandular trichome-mediated secretion, plant–insect interactions, and potential applications in integrated pest management. Non-volatile metabolites are also discussed to provide a comprehensive view of the species’ bioactive potential. Overall, *D. viscosa* emerges as a multifunctional species in which VOCs are a pivotal, though not isolated, component of a complex phytochemical system with promising applications in agriculture, pharmacology, and environmental management. While the convergence of multiple studies supports the biological potential of *D. viscosa* and its metabolites, particularly *in vitro*, the translation of these findings into *in vivo* efficacy and safety remains an important area for future research. This integrative perspective highlights the need for standardized analytical approaches and chemotype-aware studies to fully exploit the biological and biotechnological potential of this Mediterranean species.

## 1. Introduction

Volatile organic compounds (VOCs) are low-molecular-weight metabolites that play a fundamental role in plant biology, mediating interactions with the environment, shaping ecological networks, and contributing to plant defence and communication [1,2,3,4,5]. These compounds, predominantly represented by terpenoids, benzenoids, and fatty acid derivatives, participate in plant–plant, plant–insect, and plant–microbe interactions, acting as semiochemicals that influence pollination, herbivore deterrence, and the recruitment of natural enemies [1,2,3,4,6]. In particular, the allelopathic potential of *D. viscosa* has been shown to be mediated by its VOCs, which significantly alter the physiological and metabolic profiles of receiving species, highlighting their role as key drivers of plant–plant communication [6].

Beyond their ecological roles, plant-derived VOCs have attracted increasing attention for their antimicrobial, antioxidant, and antiproliferative properties, as well as for potential applications in agriculture, pharmacology, and biotechnology [7,8,9,10]. Consequently, extraction, characterization, and functional analysis of VOCs have become central themes in plant science research [2,3,8].

Within this context, Mediterranean plant species are particularly rich sources of chemically diverse VOCs, reflecting adaptation to high solar radiation, drought, and other abiotic stresses that often stimulate secondary metabolite production [11,12,13]. Among these species, *Dittrichia viscosa* (L.) Greuter (syn. *Inula viscosa*) stands out for its high plasticity and resilience, with a characteristic strong odor and sticky surface indicative of active secretion via glandular trichomes and the production of volatile and semi-volatile compounds [11,12,14]. *D. viscosa* is repeatedly reported as a rich source of essential oils, hydrosols, and headspace volatiles, dominated by mono- and sesquiterpenes whose composition varies with geographic origin, plant organ, developmental stage, and extraction method [11,12,14].

Compared with many Mediterranean Asteraceae, *D. viscosa* is distinguished by a volatile profile frequently enriched in oxygenated sesquiterpenes [6], a marked chemotypic variability across geographic regions, and the coexistence of abundant volatile and non-volatile bioactive metabolites [15]. These characteristics contribute to a highly plastic and environmentally responsive phytochemical phenotype [16] and make *D. viscosa* an attractive model for investigating how environmental factors shape VOC composition and biological activity in Mediterranean plants. Research on *D. viscosa* VOCs extends beyond chemical diversity to functional ecological roles. Accumulating evidence links these compounds to stress tolerance and interactions with herbivores and beneficial insects, contributing to ecological success [11,16,17]. Emissions are tightly linked to glandular trichome activity, identifying these structures as primary sites of VOC synthesis and secretion, thereby connecting morphological traits to chemical ecology [11,14,16]. Essential oils and other volatile fractions of *D. viscosa* also exhibit antimicrobial, antioxidant, and cytotoxic activities, underpinning potential applications in pharmaceutical and agroecological contexts [11,12,14].

However, the chemical ecology of *D. viscosa* cannot be understood through VOCs alone. These compounds operate within a broader phytochemical network that includes polyphenols, flavonoids, sesquiterpene lactones, and other specialized metabolites [11,17,18]. Evidence from plant chemical ecology suggests that biological activities often emerge from interactions among multiple metabolite classes rather than from individual compounds acting independently. VOCs primarily function as long-distance signaling molecules involved in plant–plant, plant–insect, and plant–microbe interactions, whereas non-volatile metabolites frequently provide more persistent antioxidant, antimicrobial, antiherbivore, and allelopathic effects. As highlighted by [19,20], volatile terpenoids play key ecological roles in communication and defense, while non-volatile specialized metabolites contribute to sustained protection against biotic and abiotic stresses. Moreover, evidence from phytochemical and pharmacological studies indicates that interactions among different classes of plant secondary metabolites may generate additive or synergistic effects, potentially enhancing the overall biological activity of complex plant extracts relative to isolated compounds [21,22]. Nevertheless, direct experimental evidence for such synergistic interactions in *D. viscosa* remains limited. Therefore, VOCs should be regarded as central, but not exclusive, contributors to the species’ biological activities. This perspective underscores the need for integrative approaches that simultaneously investigate volatile and non-volatile fractions, particularly considering the marked chemotypic variability and environmental modulation that characterize *D. viscosa* phytochemical profiles [11,12,23]. In addition to phytochemical richness, *D. viscosa* exhibits ecological versatility, occupying habitats from disturbed soils to coastal and semi-arid environments. This plasticity is associated with the plant’s ability to modulate secondary metabolite production, including VOCs, in response to drought, salinity, and biotic pressures, contributing to persistence and potential use in integrated pest management and environmental remediation [11,12,17]. In agroecosystems, *D. viscosa* is recognized as a reservoir of beneficial arthropods, a role plausibly mediated by VOC emissions that act as chemical cues [1,11,12]. In contaminated soils, its metabolic adaptability may influence secondary metabolite production, including volatiles, under stress, though this aspect remains underexplored [11,24]. In several plant species, abiotic stresses such as drought and heavy metal exposure have been shown to alter VOC biosynthesis and emission patterns through effects on terpenoid metabolism and stress-response pathways [25,26,27]. Heavy metals may also affect the expression of terpene synthase genes and the emission of terpenoid volatiles [28]. Given the pronounced ecological plasticity of *D. viscosa*, similar mechanisms may occur in this species; however, direct evidence linking soil contamination to changes in VOC biosynthesis or emission in *D. viscosa* is currently lacking and this relationship remains largely unexplored.

Given this multifaceted profile, *D. viscosa* represents an ideal model for exploring the interplay between volatile and non-volatile metabolites and ecological function [16]. Therefore, this review provides a comprehensive integrative synthesis of current knowledge on *D. viscosa*, which combines and critically evaluates evidence from different research domains to provide a comprehensive understanding of a topic, with a primary focus on VOCs—covering extraction methods, chemical characterization, and biological activities—and situates these volatile fractions within the broader phytochemical and ecological framework of the species to offer a holistic understanding of their functional potential and to identify key gaps for future research [29,30,31].

## 2. *Dittrichia viscosa*: Taxonomy, Morphology and Mediterranean Adaptation

**Figure 1:**
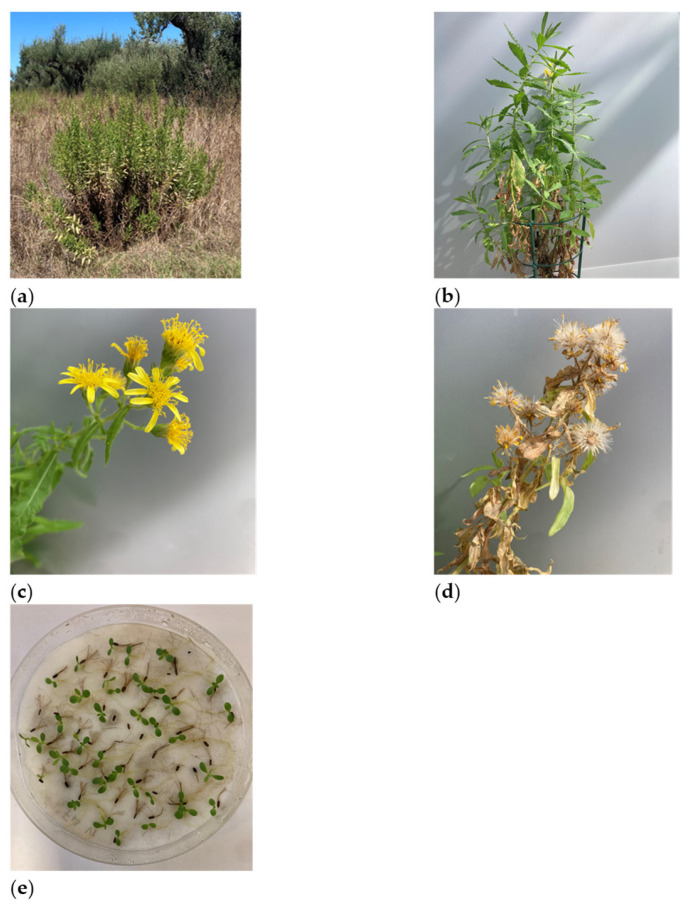
Morphological characterization and life cycle of *Dittrichia viscosa*. (**a**) Adult plant growing in its natural habitat within an olive grove near Metaponto (Basilicata, Southern Italy); (**b**) Plant cultivated under greenhouse conditions at the University of Milan (UNIMI), showing basal leaf senescence; (**c**) Close-up of inflorescences with yellow capitula at anthesis from the UNIMI *D. viscosa* accession collection; (**d**) Fruiting stage with mature dry capitula bearing a well-developed pappus for anemochorous seed dispersal, photographed in the UNIMI greenhouse collection; (**e**) Seed germination assay on moist germination paper performed in the laboratories of the University of Milan (UNIMI), showing seedling emergence and cotyledon development.

*Dittrichia viscosa* (L.) Greuter is a Mediterranean taxon whose systematic placement has long reflected the taxonomic intricacies of the Inuleae alliance within the Asteraceae (Figure 1). Historically treated within Inula as *Inula viscosa*, it is now accepted in the genus *Dittrichia*, a view supported by integrative approaches that combine morphology, micromorphology, pollen ultrastructure, and molecular insights. Across the available literature, there is a broad consensus that *D. viscosa* is closely related to other Inuleae taxa, yet several authors emphasize that genotype-by-phenotype data support delimitation at the genus level for *Dittrichia* and allied taxa. This is reinforced by phylogenetic and micromorphological work that identifies distinctive characters in fruit and pollen ultrastructure that aid circumscription within Inuleae, even though some morphological overlap persists among closely related taxa. The upshot is a nuanced taxonomy in which *D. viscosa* is accepted as the valid name, with *Inula viscosa* functioning as a historical synonym, and with delimitation anchored in a suite of characters rather than a single trait [16,32,33]. To ensure comprehensive coverage of the available literature, studies published under both *D. viscosa* and its historical synonym *Inula viscosa* were included in this review and interpreted according to the currently accepted taxonomy.

In addition to nomenclatural history, punctilious work on fruit micromorphology supports robust species discrimination within *Dittrichia* and related genera. Turkish micromorphological investigations show that *D. viscosa* possesses oblong-elliptic achenes with rugulose surface ornamentation, a pattern that helps distinguish it from close relatives such as *D. graveolens* in the Mediterranean region and neighboring areas. This diagnostic potential of achene micromorphology aligns with broader taxonomic strategies in Inuleae that integrate seed surface features with vegetative anatomy and micromorphology to improve identifications in field and herbarium material [34]. The pollen ultrastructure data, drawn from ultrastructural studies of the Inulinae, further contribute to the taxonomic toolkit, as pollen exine patterns offer comparative points among Inuleae taxa; although these features can be informative, they are most powerful when used in an integrative framework with macromorphology and molecular data [32].

Morphologically, *D. viscosa* exhibits a growth form consistent with its Mediterranean ruderal lifestyle: a perennial shrub or herb capable of producing vigorous biomass in both disturbed and more stably managed habitats. Field descriptions consistently note its broad distribution across the western and eastern Mediterranean regions, with frequent occurrence on roadsides, anthropogenically disturbed soils, and along coastal and inland transects. This ecological breadth is repeatedly highlighted as a hallmark of the species, supporting its role as a pioneer or opportunist in the Mediterranean flora matrix. The capacity to accumulate substantial biomass and to persist in disturbed substrates is repeatedly cited and forms part of the interpretation of its ecological versatility in the Mediterranean climate [16,35,36].

From a functional morphology perspective, leaf and stem anatomy and micromorphology across Inuleae, including *Dittrichia*, have been scrutinized to identify characters with taxonomic significance. In a comparative study of leaf and stem anatomy and micromorphology across several Inuleae genera, the authors analysed numerous qualitative and quantitative characters using light microscopy, SEM, and other imaging modalities. The findings indicate that while certain characters can help separate taxa, the grouping of these characters does not always coincide with current taxonomic classifications, underscoring the need for integrative approaches that combine anatomy, micromorphology, and molecular data for robust delimitation. This work demonstrates the value of vegetative anatomy and micromorphology as supportive, not definitive, lines of evidence in *Dittrichia*-related taxonomy [16,32,33].

Adaptation to the Mediterranean climate emerges as a central theme in understanding *D. viscosa*’s ecological success. The species is described as a Mediterranean ruderal that has expanded into diverse habitats, including coastal salt marsh margins, reflecting a broad tolerance to drought, heat, and salt stress typical of Mediterranean landscapes. Its distribution across Tell-style Mediterranean zones and its ability to exploit adverse habitats illustrate ecological plasticity that is characteristic of many Mediterranean broad-niche species. Comparative physiological work with related taxa indicates that *D. viscosa* employs osmotic adjustment and ion-management strategies common to halophytes, though its tolerance is not equivalent to true halophytes; it can nonetheless persist under moderate salinity and drought conditions. These adaptive traits are particularly relevant in the context of phytochemical plasticity, as drought, salinity, and other abiotic stresses are widely recognized drivers of secondary metabolite production in Mediterranean plants [25,27,37,38]. Such environmental pressures can influence the biosynthesis and emission of terpenoids and other specialized metabolites, potentially contributing to the chemotypic variability reported for *D. viscosa* across different habitats and geographic regions. Although direct evidence linking drought or salinity stress to VOC modulation in *D. viscosa* remains limited, the species’ ecological plasticity suggests that environmental conditions may play an important role in shaping its phytochemical and volatile profiles [35,39,40].

Beyond drought and salinity stress, *D. viscosa*’s adaptation includes interactions with soils and microbiota that can support its stress tolerance and survival in degraded or polluted environments. In metalliferous or polluted Mediterranean soils, *D. viscosa* has demonstrated associations with arbuscular mycorrhizal fungi (AMF) and dark septate endophytes, which are linked to metal uptake patterns and nutrient dynamics. These microbial associations mediate stress tolerance and may influence trace-element translocation within plant tissues, thereby contributing to the plant’s resilience at contaminated sites. Several studies document AMF-DSE associations across native Mediterranean plants and highlight metal accumulation patterns that often show tissue-level partitioning and variable accumulation across plant organs, with the potential for phytostabilization benefits in polluted environments. While not a hyperaccumulator, *D. viscosa*’s biomass and microbial interactions position it as a candidate for gentle remediation or stabilization strategies in semi-arid, Mediterranean soils. This microbiome-mediated component of stress tolerance and metal uptake is a recurring theme that complements the plant’s intrinsic physiological adaptations [39,41,42,43,44].

In terms of ecosystem interactions and services, *D. viscosa* has been shown to serve as an arthropod reservoir in olive groves, highlighting potential roles in natural pest control within Mediterranean agroecosystems. The pre-flowering and flowering periods attract a diverse arthropod assemblage, including predators and parasitoids, suggesting that *D. viscosa* can contribute to biological control networks, particularly under climate stress, where resilient, non-crop vegetation can sustain pest regulation services. This intertwining of plant phenology, arthropod dynamics, and agricultural practice emphasizes the species’ ecological value beyond mere biomass production, particularly in Mediterranean cropping systems that contend with climatic variability [36,44].

The taxonomic and ecological synthesis of *D. viscosa* must also acknowledge the species’ broader biogeographical context and its behavior outside the Mediterranean core. While native to the Mediterranean, *D. viscosa* has exhibited invasive behavior in non-native regions such as Australia and North America, a facet that is informative for understanding the species’ adaptive capacities and potential climate-change-driven range dynamics. This invasive footprint is contextualized within its native-range ecology, with management implications that extend to Mediterranean landscapes as climate change reshapes disturbance regimes and habitat availability. Thus, the taxonomy-adaptation narrative encompasses both the Mediterranean core and extrinsic invasion contexts, underscoring the need for regionally explicit management strategies and continued integrative research to resolve remaining taxonomic ambiguities and to refine our understanding of its ecological role across diverse Mediterranean-influenced environments [15,16,35,42].

Despite the nuances and ongoing debates across the cited sources, there is broad agreement on the Mediterranean essence of *D. viscosa*’s taxonomy and adaptation, though some nuances warrant explicit mention. First, although macromorphology consistently supports placing *D. viscosa* in *Dittrichia* and differentiating it from *Inula*, several authors stress that micromorphological and pollen characters alone are not fully diagnostic. As a result, robust delimitation relies on an integrative approach that combines leaf and stem anatomy, seed/achene micromorphology, pollen ultrastructure, and molecular phylogenetics. Second, while *D. viscosa* exhibits considerable drought and moderate salinity tolerance, its competitive edge against true halophytes under high salinity is limited, which translates into habitat partitioning at salt marsh margins rather than the displacement of specialized halophytes. Third, the species’ role in phytoremediation and metal-contaminated soils, while promising for stabilization, remains conditional on biomass production, soil chemistry, and microbial associations, all of which can vary across the Mediterranean gradient. Finally, there is some geographic nuance in micromorphological characters (e.g., achene ornamentation) that can aid regional identification but should be used in combination with other data to avoid misinterpretation in areas with closely related taxa.

To advance a cohesive understanding of *D. viscosa*, future research should pursue integrative taxonomic frameworks that couple comprehensive micromorphology, vegetative anatomy, and multilocus phylogenetics across the Inuleae, including *Dittrichia*, *Inula*, *Limbarda*, and *Pulicaria*, to clarify species boundaries and phylogenetic relationships. In situ and controlled experiments comparing *D. viscosa* with co-occurring halophytes (e.g., *Inula crithmoides*) under varying salinity and drought regimes would sharpen our understanding of niche differentiation and mechanism-based adaptation to Mediterranean wetlands [32,33,34,35,36]. Longitudinal studies on AMF and DSE associations across the Mediterranean gradient would illuminate microbe-driven components of stress tolerance and metal uptake. On the applied side, phytostabilization and gentle remediation studies on contaminated Mediterranean soils could leverage *D. viscosa*’s biomass-microbiome partnerships while evaluating ecological risks and ecosystem-service trade-offs in semi-arid ecosystems. Taken together, an integrative battery of taxonomic, ecological, physiological, and microbiome-centered approaches will yield a more precise portrait of *D. viscosa* within the Mediterranean landscape and beyond [15,30,31,32,33,34,41,42,43,44].

## 3. Traditional and Ethnobotanical Uses of *Dittrichia viscosa*

Beyond its well-documented phytochemical complexity and biological activities, *D. viscosa* has a long history of use in traditional Mediterranean medicine, where it has been employed for various therapeutic purposes. Ethnobotanical surveys conducted across Mediterranean regions, including North Africa and the Middle East, document the use of *D. viscosa* in folk medicine to treat skin disorders, inflammatory conditions, infections, and metabolic ailments [45,46].

Topical applications are among the most frequently documented uses: decoctions, poultices, or macerated plant material have traditionally been applied to wounds, burns, and dermatological infections, reflecting the plant’s perceived antiseptic and healing properties. These uses are consistent with the antimicrobial and anti-inflammatory activities later demonstrated *in vitro* for extracts and essential oils of *D. viscosa* [47,48].

In addition to external applications, oral preparations such as infusions and decoctions have been used in traditional medicine to treat gastrointestinal disorders, diabetes-related symptoms, and respiratory conditions [46,49]. The reported antidiabetic and enzyme-inhibitory activities of *D. viscosa* extracts, particularly against α-amylase and α-glucosidase, provide a pharmacological basis that partially supports these traditional uses [49]. Furthermore, the plant has been traditionally used as a natural insect repellent and protective agent against pests, a practice that aligns with its high levels of volatile terpenoids and its ecological role in plant–insect interactions [6,16]. This traditional repellent function is scientifically supported by the plant’s rich emission of volatile terpenoids, which have been documented to mediate complex ecological defenses and interspecific interactions [6]. In some rural contexts, its sticky exudates have also been associated with protective functions against herbivores and insects, reinforcing its perception as a defensive plant species.

Despite this broad spectrum of traditional applications, it is important to note that ethnomedicinal uses are often based on empirical knowledge and are not always supported by standardized clinical evidence. Moreover, variability in phytochemical composition across regions and plant parts may influence efficacy and safety, highlighting the need for controlled pharmacological and toxicological studies to validate traditional claims [46].

Overall, the ethnobotanical relevance of *D. viscosa* provides a valuable framework for interpreting its pharmacological potential, as many traditional uses are increasingly corroborated by modern phytochemical and biological investigations, supporting its role as a promising source of bioactive compounds.

In Italy, *D. viscosa* has been traditionally integrated into rural and agro-pastoral practices, although specific ethnobotanical documentation remains comparatively limited and often overlaps with broader Mediterranean knowledge frameworks. Within this context, its medicinal use is consistent with ethnobotanical reports from other regions, where the plant has been employed in topical preparations (e.g., poultices and decoctions) for the treatment of wounds, skin infections, and inflammatory conditions, reflecting empirically recognized antiseptic and healing properties later supported by its antimicrobial and anti-inflammatory activities [46,47,48]. In Italian rural traditions, particularly in central and southern areas, *D. viscosa* has also been used in veterinary practices, where fresh plant material was applied to treat lesions in livestock and as a repellent against ectoparasites, a function that can be mechanistically linked to its terpene-rich secretions and glandular trichome activity, as well as to its ecological role in plant–insect interactions [6,16]. This repellent function extends to its traditional use in domestic and storage environments, where the plant was placed to deter insects, consistent with its documented volatile-mediated bioactivity [6].

**Figure 2:**
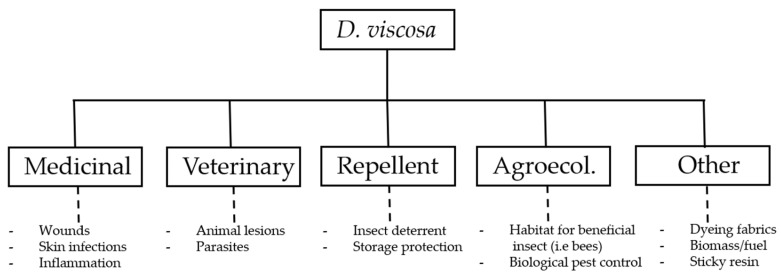
Schematic representation of the traditional uses of *D. viscosa* in Mediterranean contexts with emphasis on Italy. The species has historically been used for medicinal purposes, including treating wounds, skin infections, and inflammatory conditions, and in veterinary practice for managing animal lesions and parasites. Its volatile-rich and sticky exudates underpin its traditional use as a natural insect repellent in domestic and storage environments. In agroecosystems, *D. viscosa* supports beneficial arthropods that provide multiple ecosystem services. Its flowers offer nectar and pollen resources for pollinators (e.g., *Apis mellifera* and *Bombus* spp.), enhancing pollination. The plant also provides resources and refuge for predators and parasitoids (e.g., ladybirds, lacewings, spiders, and braconid wasps), which contribute to biological pest control. In particular, *D. viscosa* supports the parasitoid wasp *Psyttalia concolor*, a natural enemy of the olive fruit fly (*Bactrocera oleae*), thereby contributing to the biological control of this key olive pest. Additional minor uses include its application as a source of yellow–brown natural dyes and as biomass or resinous material in rural practices.

Furthermore, in Mediterranean Italian agroecosystems, especially olive groves, *D. viscosa* has long been present as a spontaneous component of the vegetation, and recent ecological studies highlight its role as a reservoir of beneficial arthropods, including predators and parasitoids, thereby contributing to natural pest regulation and reinforcing its functional relevance within traditional low-input agricultural systems [36,44]. In addition to these better-documented uses, ethnobotanical knowledge in Italy also indicates a minor application of *D. viscosa* as a source of natural dyes, with aerial parts yielding yellow to brown pigments likely associated with flavonoids (Figure 2).

## 4. The Ecological Significance of Viscosity in *Dittrichia viscosa*: Glandular Trichomes as Bio-Factories

**Figure 3:**
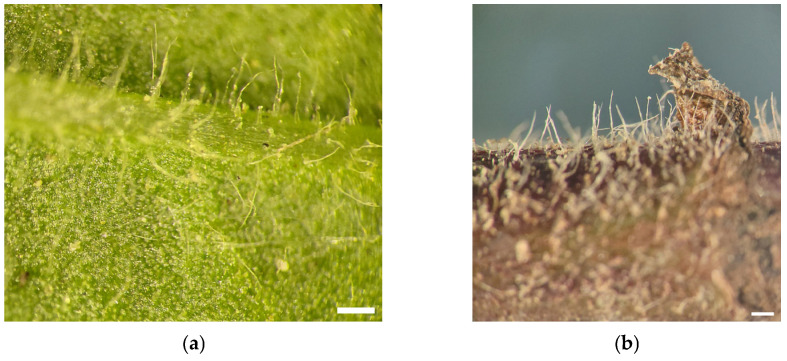
Morphological characterization of trichomes in *Dittrichia viscosa*. Fresh apical stem segments and associated leaves collected from field-grown plants in Montalto Uffugo (Cosenza, Italy) were examined immediately after sampling using a Leica MZ12 stereomicroscope (Leica Microsystems GmbH, Wetzlar, Germany), without fixation or further sample preparation. (**a**) Leaf surface: The micrograph highlights the coexistence of two distinct types of hairs: glandular trichomes, characterized by shorter stalks and rounded secretory heads responsible for the plant’s resinous exudate, and non-glandular trichomes, which appear as elongated, translucent filaments providing physical protection. (**b**) Stem surface: A profile view showing a high density of non-glandular trichomes. These long, unbranched hairs form a protective barrier over the epidermis, interspersed with smaller glandular structures visible at the base of the indumentum. Scale bar: 500 μm.

In *D. viscosa*, surface viscosity is a conspicuous and ecologically meaningful trait that has attracted interest as a possible manifestation of glandular trichome activity (Figure 3). Rather than attributing stickiness merely to passive cuticular residues, an integrated view situates leaf and stem viscosity within a secretory framework driven by glandular trichomes (GTs). Across diverse taxa, GTs are well established as biosynthetic and storage organs for a broad repertoire of secondary metabolites, and their secretions often give rise to viscous, adhesive surface films that mediate a suite of ecological interactions. Converging lines of evidence from anatomy, ultrastructure, histochemistry, and ecological chemistry thus support the interpretation that the viscosity observed on *D. viscosa* surfaces is, at least in large part, an emergent property of GT secretion and organization, rather than a byproduct of non-secretory leaf surface chemistry alone [31,50,51].

A generalizable glandular-trichome framework underpins the GT–viscosity link in *D. viscosa*. GTs are repeatedly described as true cellular factories capable of producing, storing, and secreting diverse secondary metabolites, including lipids, terpenoids, polysaccharides, and phenolics. Their strategic location at the epidermis and their secretion through subcuticular spaces or cuticular pores position GT-derived products as primary determinants of leaf and stem surface chemistry and physical properties. Within Asteraceae, GT architecture, ranging from long-stalked to multiseriate heads, correlates with secretion modes (granulocrine versus eccrine) and with classes of metabolites, which in turn shape the ecological function of the exudates. This integrated conceptual model is applicable to *Inula viscosa* and related taxa, in which secretory activity has been shown to involve multiple chemical components beyond lipophilic secretions alone [31,51,52,53]. The accumulation of viscous material in the subcuticular space and its subsequent deposition on organ surfaces aligns with a GT-based secretion ecology that can produce a sticky surface capable of mediating interactions with herbivores, pathogens, and abiotic environmental factors [2,31,51].

Chemically, GT secretions in the Asteraceae are complex, and their composition frequently includes lipids, terpenoids, polysaccharides, and proteins. Such components are known to contribute to surface stickiness and viscosity and to possess ecological activities such as antimicrobial, antifungal, and antifeedant effects. Documented GT secretions in *Heterotheca* and related taxa contain sesquiterpene lactones and diterpenes with defensive roles, illustrating how GT-derived chemical inventories translate into ecological outcomes that include deterrence of herbivores and protection against microbial colonization. Although these examples come from related taxa, they illuminate a plausible chemical substrate for *D. viscosa* viscosity, wherein terpenoids and lipophilic compounds are often major constituents of GT secretions that contribute to surface tackiness and ecological defence [31,51,54,55,56]. In *Inula viscosa* specifically, essential oils and GT secretions with antimicrobial activities have been reported, with secretion quantity and quality modulated by GT structure, tissue location, and developmental stage, further supporting a GT-centered basis for surface viscosity as a context-dependent ecological trait [31,35,51].

An ultrastructural and histochemical perspective reinforces the mechanistic interpretation. GT heads and stalks typically comprise secretory cells rich in organelles such as chloroplasts, mitochondria, ER, Golgi, and vesicles, reflecting robust biosynthetic and secretory capacity. Secretions accumulate in subcuticular spaces and exit via cuticular pores, a pattern consistent with sticky exudates observed on plant surfaces [31]. Histochemical analyses commonly reveal a mixture of lipids, polysaccharides, proteins, and polyphenolic compounds in GT secretions, sometimes accompanied by alkaloids in certain taxa. This multi-component secretion aligns well with a viscosity that serves multifaceted ecological roles, including defense against biotic stress (herbivores, pathogens) and modulation of plant–microbe and plant–insect interactions through a biochemical cocktail produced by GTs. The broader GT literature in Asteraceae corroborates that GT products are organ- and tissue-specific, with flexible secretion across leaves, stems, inflorescences, and fruits, all contributing to the surface viscosity profile of the plant [51,52,53].

Ecologically, the viscosity of *D. viscosa* surfaces can be interpreted as part of a defence and interaction strategy orchestrated by GTs (Table 1). The GT-driven secretion system equips the plant with a viscous matrix that can deter herbivory directly via antifeedant compounds and indirectly by trapping or repelling pests. The extreme stickiness of GT secretions is often attributed to terpenoids and related lipophilic compounds, which can serve as physical and chemical deterrents. The VOCs synthesized within these glandular structures not only contribute to surface viscosity but also serve as potent allelopathic agents; a metabolomic approach has indeed revealed how these emissions can suppress the growth of competing vegetation by interfering with primary and secondary metabolism [6]. The ecological literature on GTs across Asteraceae and related groups demonstrates antifeedant activities and antimicrobial properties of GT secretions, providing a chemical-ecological rationale for viscosity as a defensive trait [16]. This perspective is consistent with observations in *D. viscosa* and its relatives, where GT secretions are associated with defence and ecological plasticity across habitats, including Mediterranean and disturbed environments. Consequently, GT-produced viscosity likely contributes to *D. viscosa*’s invasiveness and stress tolerance by shaping interactions with herbivores, pollinators, and microbial communities [35,55,56].

The discussion of *D. viscosa* in ecological and chemical terms also points to broader biotechnological implications of GTs as bio-factories. GTs are attractive targets for metabolic engineering because they naturally accumulate high concentrations of diverse metabolites and present a relatively accessible source of enzymes, transcripts, and small molecules for analytical and biotechnological exploitation [31]. This aligns with a growing consensus that glandular trichomes can serve as scalable plant-based platforms for producing terpenoids and other valuable compounds, including potential bioadhesives and antimicrobial agents. In this sense, the GT-mediated secretion system in *D. viscosa* not only explains its viscosity as an ecological trait but also highlights a potential route for bioengineering applications in sustainable agriculture and bioproduct development, leveraging GTs to produce and deploy functional metabolites in crop protection, biopolymer adhesives, and antimicrobial formulations [35,50,56].

It is worthwhile to acknowledge nuance and ongoing uncertainty within the GT literature. Although a consistent pattern emerges in which GTs drive secretions that underpin surface viscosity and ecological function, the precise chemical profile of a given species’ GT exudate is tissue- and context-dependent. Tissue type (leaf, stem, or reproductive organs), developmental stage, and environmental conditions (drought, salinity, herbivory pressure) can shift the relative abundance of lipid, polysaccharide, protein, and terpenoid components in the secretion. Therefore, while the GT-centric interpretation of *D. viscosa* viscosity is well-supported, exact molecular correlates may vary and require targeted chemical profiling to map specific metabolites to ecological outcomes in particular tissues and habitats [31,35,51].

From a practical standpoint, this perspective implies several avenues for further study. For ecology, it would be informative to quantify GT density across organs and to correlate GT secretory output with measured viscosity, organ hydration status, and herbivore pressure under different environmental scenarios. Such work would help disentangle the contribution of GT secretions versus non-secretory cuticular lipids in shaping surface stickiness. For chemistry and pharmacology, broader profiling of GT secretions in *D. viscosa* across tissues and developmental stages could reveal specific antimicrobial or antifeedant compounds with ecological and pharmacological relevance, aligning with reports from Inula and *Dittrichia* relatives that GTs host rich chemical inventories with ecological activities [31,35,56]. For biotechnology, insights into GT development and secretion pathways could inform strategies to bioengineer GT-like factories in crop species or to exploit plant-derived adhesives and antimicrobial compounds in sustainable applications, building on the conceptual and empirical foundations that GTs are robust, versatile bio-factories for secondary metabolites [31,50,56].

## 5. Volatile Organic Compounds: Composition and Variability in *Dittrichia viscosa*

Volatile organic compounds (VOCs) produced by *D. viscosa* display pronounced chemical diversity and dynamic variability that are tightly linked to genotype, geography, plant organ, developmental stage, and the extraction or sampling method used. Across the Mediterranean, essential oils (EOs) of *D. viscosa* are consistently enriched in oxygenated sesquiterpenes, yet the exact major and minor components show region-specific patterns: caryophyllene oxide, 1,8-cineole, α-muurolol, and terpenoid lactones frequently appear as prominent lipophilic constituents, but populations from Sardinia, Croatia, Spain, Algeria, and other locales can differ markedly in the relative abundances of isocostic acid, fokienol, borneol, bornyl acetate, and other terpenoids [45,60,61]. This regional chemotypic diversity underpins substantial intra- and inter-population variability in EO composition and, by extension, in biological activity, because the lipophilic fraction and the hydrosol (the aqueous distillate) partition volatiles differently and thus present distinct chemical landscapes and bioactivities [45,60,61]. In Croatian samples, for example, the total EO yield is modest (approximately 0.09% on a dry-weight basis), yet 1,8-cineole and caryophyllene oxide remain detectable lipophilic markers, while the hydrosol is enriched in polar volatiles such as p-menth-1-en-9-ol and cis-sabinene hydrate, illustrating a clear composition dichotomy between the two fractions arising from the same botanical source [60]. The divergence between essential oil and hydrosol compositions is a recurrent theme across studies and is attributed to solubility and partitioning effects that separate lipophilic and hydrophilic volatiles during distillation and phase separation [60,61].

Extraction method and harvest context further modulate VOC profiles. Differences in steam-distillation, ultrasonic-assisted extraction, and solvent extraction, combined with the plant’s provenance and phenological stage, yield distinct chemotypes and alter the balance between sesquiterpene-dominated and monoterpene-rich oil classes. In this regard, Croatian and broader Mediterranean datasets illustrate that major components such as 1,8-cineole, caryophyllene oxide, borneol, and borneyl acetate can fluctuate in relative abundance across regions and methods, underscoring the need for standardized approaches when comparing chemotypes and assessing bioactivity [60,61,62]. The literature also notes that the compositional landscape of *D. viscosa* EO is enriched in sesquiterpene lactones and other secondary metabolites whose prevalence varies with soil, climate, and phenology, reinforcing the view that chemotype identity is multi-factorially determined rather than monolithic across the species range [60,61,62].

The extraction methodology itself represents an important source of variability in the reported VOC composition of *D. viscosa*. Hydrodistillation remains the most widely employed technique for essential oil isolation, providing comprehensive profiles of mono- and sesquiterpenes, although prolonged heating may promote the degradation or loss of highly volatile constituents. In contrast, headspace solid-phase microextraction (HS-SPME) enables the analysis of volatiles released under near-native conditions without thermal processing, making it particularly suitable for profiling highly volatile compounds rather than the complete essential oil composition [63,64]. Solvent- and ultrasound-assisted extraction generally recover a broader spectrum of less volatile terpenoids and other secondary metabolites, with extraction efficiency strongly influenced by solvent polarity and operating conditions. Consequently, differences observed among published VOC profiles may reflect not only geographical origin and plant organ, but also methodological biases associated with the extraction and analytical approaches employed, highlighting the need for standardized protocols to facilitate comparisons among studies [46,60,62].

Additional evidence from Algerian, Turkish, and Spanish populations further highlights the pronounced chemotypic variability of *D. viscosa*. While some populations are characterized by high abundances of polygodial, phytol, intermedeol, and fokienol, others display profiles enriched in monoterpenes or oxygenated sesquiterpenes [61,62]. Similar variability has also been reported in headspace analyses, where the relative abundance of mono- and sesquiterpenes changes according to geographic origin and plant organ, reinforcing the dynamic nature of the species’ volatile metabolome [60,65].

Functionally, this chemotypic and methodological variability translates into divergent biological activities. The essential oil and hydrosol fractions exhibit antimicrobial effects that are not uniform across populations or fractions; activity profiles depend on the prevailing VOC suite, with certain chemotypes showing stronger antibacterial or antibiofilm effects, potentially owing to higher contents of active constituents such as caryophyllene oxide, 1,8-cineole, or borneol, among others [60,62,63]. Some studies report antiproliferative or antiphytoviral activities for *D. viscosa* volatiles, with hydrosol- and EO-specific effects indicating differential mechanisms and targets; however, the precise active constituents and their synergistic relationships remain to be fully delineated and replicated across regions [60,62,63]. Moreover, ecological and agricultural implications—such as phytotoxicity and allelopathic interactions driven by VOCs-appear contingent on chemotype and environmental context, illustrating how VOC variation can influence plant–plant and plant–pathogen interactions in situ [16,63,64].

From a practical standpoint, the pronounced VOC diversity observed in *D. viscosa* cautions against broad generalizations about the species’ essential oil chemistry or its bioactive potential. For applications in antimicrobial formulations, natural pesticides, or crop protection strategies, sourcing must be chemotype-characterized and regionally representative to ensure reproducible efficacy. The duality of EO and hydrosol composition further suggests complementary utilities: EO fractions may be prioritized for lipophilic antimicrobial or insecticidal activities, whereas hydrosols could offer polar, water-soluble bioactives with distinct antimicrobial or phytochemical effects. Finally, integrating cross-regional datasets with standardized extraction and analytic protocols would enable more robust chemotype mapping, clearer links between specific VOCs and bioactivities, and better-informed exploitation of *D. viscosa* as a source of eco-friendly bioactive compounds [60,61,62,63]. A comparative overview of the main VOC profiles reported for *D. viscosa* across different geographical regions, plant organs, extraction procedures, analytical approaches, and associated biological activities is provided in Table 2.

## 6. Phytochemical Profile of *Dittrichia viscosa*: Not Only Volatiles

Phytochemical profiling of *D. viscosa* reveals a plant that is unusually rich in both volatile and non-volatile phytochemicals, with concerted evidence across essential oils (EOs), hydrosols, flavonoids and hydroxycinnamic acids in leaf- and root-derived extracts, and a spectrum of headspace volatiles released by the plant. The literature consistently demonstrates that the chemical landscape of *D. viscosa* is markedly chemotype-dependent, reflecting geographic origin, plant part, developmental stage, and extraction methodology, yet it converges on a core pattern: a terpene-heavy EO punctuated by oxygenated terpenes and sesquiterpenoids, alongside polyphenol-rich extracts that carry potent antioxidant and enzyme-inhibitory activities. This integrated view emerges from cross-referenced analyses of GC–MS, GC–FID/MS, HS-SPME, HPLC-DAD/ESI-MS, and UHPLC-MS profiling conducted on leaves, stems and roots from different Mediterranean regions and across multiple extraction workflows.

Although volatile organic compounds (VOCs) and essential oil constituents represent major components of the phytochemistry of *D. viscosa* and have been discussed in detail in Section 5, they represent only one aspect of the species’ chemical diversity. Previous studies have shown that both the emitted VOC profile and the composition of the essential oil are dominated by mono- and sesquiterpenes, although they vary according to geographic origin, plant organ, and analytical or extraction methodology [60,61,62,63,64,65].

However, the biological and pharmacological properties of *D. viscosa* cannot be explained by VOCs alone, as numerous non-volatile metabolites substantially contribute to the species’ bioactivity.

When addressing non-volatile phytochemicals, leaf- and root-derived extracts reveal a complementary and biologically meaningful dimension of *D. viscosa* chemistry. Ethyl acetate (EtOAc) and methanolic extracts of Moroccan and North African material yield high total polyphenol contents and substantial flavonoid pools, with HPLC-DAD/ESI-MS analyses detecting hydroxycinnamic acids, dicaffeoylquinic acids, rosmarinic acid, and a suite of flavonoids; these polyphenols underpin strong antioxidant activities in DPPH, ABTS, and FRAP assays and contribute to enzyme inhibition relevant to metabolic disorders, notably α-amylase and α-glucosidase inhibition, underscoring antidiabetic potential [48,49,68,69]. The antidiabetic potential of *D. viscosa* is mainly supported by *in vitro* α-amylase and α-glucosidase inhibition assays performed on crude leaf extracts, which have demonstrated significant enzyme inhibitory activity [48]. More recently, similar activities have also been reported for the essential oil, with the experimental findings further supported by molecular docking analyses [67]. However, evidence based on isolated compounds remains limited, and further *in vivo* studies are required to validate these findings. Algerian ethanolic extracts likewise reveal a rich phenolic landscape with hydroxycinnamic acids and flavonol derivatives as major constituents, supporting cytotoxic and other bioactivities; UHPLC-DAD-ESI/MS analyses document 17–21 polyphenolic compounds in leaf extracts, with hydroxycinnamates and chlorogenic derivatives often prominent [47,49,70]. Root tissues add another facet: hydromethanolic root extracts and corresponding essential oils show distinct polyphenolic signatures (e.g., hesperin, naringenin, kaempferide, tangeritin) and terpenoid predominance (β-acorenol and related esters) in the EO, with robust antioxidant activity and notable cytotoxic and antimicrobial properties, implying organ-specific partitioning of terpenoids and polyphenols that broadens potential applications from nutraceuticals to pharmaceuticals [48,66].

Beyond polyphenols and flavonoids, *D. viscosa* is also recognized as an important source of sesquiterpene lactones, a characteristic class of specialized metabolites widely distributed in the Asteraceae. Compounds such as tomentosin, inuviscolide, and related sesquiterpene derivatives have been identified in different organs of the plant and have attracted considerable attention because of their antimicrobial, anti-inflammatory, cytotoxic, and antiproliferative activities [71,72]. These metabolites constitute an important non-volatile component of the phytochemical profile of *D. viscosa* and significantly contribute to its pharmacological potential.

The integrated pattern across plant extracts, volatile emissions, and essential oil fractions indicates that non-volatile polyphenols and volatile terpenoids function in tandem to shape *D. viscosa*’s bioactivity profile [48,49]. The EtOAc leaf extracts, rich in polyphenolics and with strong antioxidant capacity, dovetail with essential oil fractions that are terpenoid-dense and bioactive against microbial targets and digestive enzymes. This synergy aligns with broader reviews and systematic treatments of the Inuleae-Inulinae clade, where dicaffeoylquinic acids, rosmarinic acid, and various hydroxycinnamate esters are repeatedly found alongside sesquiterpenoids and other terpenoids as major metabolite classes, collectively accounting for antioxidant, antimicrobial, anti-inflammatory, and antiproliferative activities reported for *D. viscosa* and related taxa [71,72]. The cross-referencing of leaf, root, and VOC profiles and essential oil compositions across regionsalso highlights a recurring theme [47]: chemotype diversity is a hallmark of *D. viscosa*, with major constituents shifting by country, population, plant part, and extraction method, yet the overarching narrative remains one of a terpene-rich volatile architecture coupled with polyphenol-driven non-volatile bioactives [60,61,62,66,70].

A particularly informative aspect of the phytochemistry of *D. viscosa* concerns the biological activities associated with its terpenoid constituents. Several studies link EO composition to antimicrobial activity and enzyme inhibition relevant to metabolic regulation; in some datasets, costic acid and other terpenoid derivatives are singled out as active components with antimicrobial or cytotoxic potential, while monoterpene VOCs, including α-pinene, β-myrcene, and p-cymene, contribute to the ecological and pharmacological relevance of the species, non-volatile terpenoids also appear to play an important role in its biological activities. The work exploring terpenoid-rich fractions of *Inula viscosa* for anticancer activity against lung cancer cells further exemplifies how organostructure–dependent terpenoids (e.g., lupeol, caryophyllene oxide, dearomatized terpenoids) can exert cytotoxic effects while sparing normal cells, highlighting the therapeutic potential of the diverse terpenoid repertoire of *D. viscosa* and the importance of considering both volatile and non-volatile constituents when evaluating its biological properties [66,72].

In sum, the phytochemical portrait of *D. viscosa* is best understood as a chemotype-laden mosaic in which VOC emissions, essential oil constituents, and hydrosol constituents collectively define its volatile chemical profile, whose composition varies according to geographic origin, plant organ, and analytical methodology, while leaves and roots contribute rich pools of non-volatile polyphenols and flavonoids that confer potent antioxidant and enzyme-inhibitory activities (Table 3A–D) [47,49]. The convergence of GC–MS, HS-SPME, and UHPLC-MS data across multiple studies substantiates a cohesive view of *D. viscosa* as a multifunctional phytochemical reservoir with clear translational potential for antimicrobial, anti-inflammatory, antidiabetic, and anticancer applications [60,61,62]. Yet the nuances are nontrivial: regional chemotypes can invert the relative dominance of monoterpenes versus oxygenated sesquiterpenes in EO composites, and plant part–dependent partitioning can shift the balance between volatile and non-volatile metabolites [66,70], a nuance that calls for standardized, cross-regional chemotype characterization to better harness the plant’s bioactive repertoire in targeted applications [68,72].

## 7. Biological Activities of *Dittrichia viscosa* and Its Compounds: Antioxidant, Antibacterial, and Antitumor Properties

As previously reported, *D. viscosa* is characterized by a chemical repertoire that includes a rich array of polyphenols, flavonoids, terpenoids, and, notably, sesquiterpene lactones such as tomentosin and inuviscolide. An accumulating body of evidence, drawn from studies on plant extracts, essential oils, hydrosols, and isolated constituents, converges on three broad bioactivities of substantial interest: antioxidant capacity, antibacterial potential, and antiproliferative (antitumor) effects. Across independent laboratories and diverse geographic regions, the literature reveals both consistent themes and nuanced variations that reflect phytochemical diversity, extraction methods, and the model systems tested. Here, we synthesize these strands into a cohesive narrative, explicitly linking claims to supporting references in IEEE style and noting areas of agreement, as well as any disagreement or nuance, where present. Collectively, the available literature indicates that *D. viscosa* is a multifunctional medicinal species whose biological activities arise from the complementary action of multiple classes of secondary metabolites rather than from a single bioactive compound. Phenolic-rich methanolic and ethanolic extracts consistently exhibit strong antioxidant activity together with anti-inflammatory, antiglycation, and α-amylase/α-glucosidase inhibitory properties, which have been mainly attributed to caffeoylquinic acid derivatives, flavonoids, and other phenolic constituents [48,49,73]. In contrast, essential oils and hydrosols are primarily associated with antibacterial and antifungal activities, while also displaying antioxidant, anti-inflammatory, antiproliferative, and enzyme-inhibitory effects that are largely related to their terpene-rich composition [60,67]. Among the individual metabolites, the sesquiterpene lactones tomentosin and inuviscolide have emerged as key contributors to the antitumor potential of *D. viscosa*, inducing apoptosis, modulating oxidative stress, and interfering with cell-cycle progression in several *in vitro* models [46]. Overall, these findings reinforce the pharmacological and agricultural relevance of *D. viscosa* while also highlighting the considerable variability among studies, largely driven by differences in extraction procedure, plant organ, developmental stage, chemotype, and geographical origin. Future research should therefore prioritize standardized extraction protocols and integrated metabolomic and biological approaches to establish clearer relationships between phytochemical composition and biological activity, thereby facilitating the development of *D. viscosa*-derived products for pharmaceutical and agricultural applications.

### 7.1. Antioxidant Activity and Its Phytochemical Basis

A central pattern across studies is that *D. viscosa* exhibits robust antioxidant activity that closely tracks its polyphenol and flavonoid content. Methanolic leaf extracts consistently show high total phenolics and flavonoids, correlating with strong radical-scavenging capacity in standard assays (e.g., DPPH and ABTS) and antiglycation effects. These observations are supported by multiple investigations focusing on leaf extracts, with the methanolic preparation frequently outperforming aqueous counterparts, presumably due to greater solubility of phenolics and related antioxidants [48,67,69,72]. The emphasis on caffeoylquinic acid derivatives, particularly 3,4-dicaffeoylquinic acid, as significant contributors to antioxidant activity further solidifies the phytochemical basis for this activity, with leaf-derived extracts repeatedly highlighted for their high polyphenol content and associated radical-scavenging potential [48,72].

Beyond crude extracts, volatile and hydrophilic fractions also contribute to antioxidant capacity. Essential oils of *D. viscosa*, while typically studied for antimicrobial or antiproliferative actions, have shown assay-dependent antioxidant effects, attributed to monoterpenes and phenolic constituents detectable in the oil or in hydrosol fractions (e.g., borneol-related compounds, 1,8-cineole, and related terpenoids [60,62,74]. Comparative assessments indicate that essential oils can display meaningful, albeit sometimes lower or more variable, antioxidant performance relative to crude methanolic extracts, underscoring the importance of preparation type in interpreting antioxidant potential [60,62,67].

Mechanistically, the antioxidant effects are attributed to hydrogen- or electron-donating polyphenols and terpenoids that scavenge reactive oxygen species (ROS) and modulate redox status in cellular contexts. While most evidence is *in vitro*, the mechanistic framing is consistent with polyphenol- and terpenoid-mediated redox modulation and scavenging, which may have implications for cancer biology where oxidative stress intersects with signaling and cell fate decisions [48,62,75]. Synthesis of the literature thus supports a robust antioxidant profile for *D. viscosa*, appreciably influenced by plant part and solvent, with leaf methanolic extracts typically providing the strongest *in vitro* antioxidant responses [48,72].

### 7.2. Antibacterial Activity and Spectrum of Activity

*D. viscosa* exhibits broad antibacterial activity across several preparations, including methanolic, ethanolic, and aqueous extracts, as well as essential oil and hydrosol fractions [60,62,69]. The antimicrobial spectrum commonly includes Gram-positive bacteria such as *Staphylococcus aureus* and *Bacillus subtilis*, and Gram-negative organisms like *Escherichia coli* and *Pseudomonas* species, with potency varying by extract type and tested organism [67,76,77]. Notably, essential oils and hydrosols contribute distinct antibacterial profiles that reflect their volatile composition, including monoterpenes and sesquiterpenes such as 1,8-cineole and caryophyllene oxide, which have been associated with antimicrobial activity in several *D. viscosa*-based studies [60,62,67].

The variability in antibacterial efficacy across studies is plausibly linked to chemotypic diversity and geographic origin, as well as methodological differences (disc diffusion *vs.* MIC/MBC). For instance, essential oils from different Algerian populations or Turkish/western Mediterranean cohorts show differing spectrums and magnitudes of inhibition, consistent with reported chemotypes and the presence of compounds such as polygodial, intermedeol, phytol, and borneol derivatives that shape antimicrobial activity [60,61,71,78]. Hydrosols offer complementary, sometimes modest, antimicrobial effects and may harbor phenolic acids (e.g., 3,4-dihydroxybenzoic acid) contributing to the observed activity in hydrosol fractions [60].

In addition to direct antimicrobial effects, several studies note potential synergistic interactions between *D. viscosa* preparations and conventional antibiotics, suggesting that plant-derived matrices may enhance antimicrobial efficacy or enable dose-sparing strategies [60,61,62]. Such synergy has been discussed in relation to leaf matrices rich in tannins, flavonoids, saponosides, sterols, and triterpenes, which can interact with bacterial targets and membrane components [74,79,80]. Taken together, the antibacterial literature supports a consistent, though composition-dependent, antimicrobial potential for *D. viscosa*, with stronger activity typically arising from methanolic extracts and essential oils, and with chemotype and origin contributing to inter-study variability [67,76,77].

### 7.3. Antitumor (Antiproliferative) Properties and Mechanisms

Across multiple studies, *D. viscosa* and its constituents—particularly its sesquiterpene lactones tomentosin and inuviscolide—emerge as notable antiproliferative agents *in vitro*. Tomentosin, a prominent SL in *D. viscosa*, has demonstrated cytotoxic effects in various cancer cell lines, including leukemia, cervical cancer, and other tumor models, with evidence of apoptosis induction, ROS generation, cell-cycle arrest, and disruption of proliferation pathways [60,62,69]. Inuviscolide also contributes to cytotoxic outcomes, and in combination, these SLs in *D. viscosa* extracts correlate with antiproliferative effects across tested cancer cell panels [67,74,81].

The mechanistic landscape for sesquiterpene lactones (SLs) centres on covalent modification of protein cysteine residues, activation of intrinsic apoptotic programs (caspases, Bcl-2 family regulation), ROS-mediated damage, and interference with cell cycle progression and angiogenesis. Reviews of SLs highlight proapoptotic actions and potential proteasome-inhibitory effects as core antitumor mechanisms, with additional context in signalling pathways and tumor microenvironment modulation. This mechanistic breadth is echoed in discussions of tomentosin- and inuviscolide-containing extracts and their apoptotic signaling signatures, including evidence of ROS involvement and caspase activation in various tumor models [74,81].

*In vitro* studies of *D. viscosa* volatiles and terpenoid-rich fractions also report antiproliferative activity against cancer cell lines, including HeLa, HCT116, and U2OS, with reductions in viability and indications of apoptosis. Mechanistic hints from these reports include ROS perturbations and activation of intrinsic apoptotic pathways, aligning with SL-driven cytotoxic paradigms while acknowledging that the full spectrum of targets remains to be defined for different tumor models [60,67,74].

Overall, there is robust *in vitro* support for antitumor activity of *D. viscosa* and its key SLs, especially tomentosin and inuviscolide, with consistent themes of apoptosis induction and oxidative stress involvement [60,62,69]. While *in vivo* data are more limited relative to *in vitro* findings, the convergence of multiple studies and reviews across plant parts and extracts strengthens the view that *D. viscosa* represents a promising source of antiproliferative agents, potentially suitable for combination strategies or as lead compounds for further development. Nevertheless, the translation to *in vivo* efficacy and safety remains an important area for future work [67,74,81].

### 7.4. Nuances, Regional Variation, and Integration of Findings

A recurring theme across the antioxidant, antibacterial, and antitumor studies is regional phytochemical variability. Geographic origin, environmental conditions, and extraction methods markedly influence the chemical profiles of *D. viscosa*, which in turn modulate observed bioactivities. Mediterranean, North African, and adjacent regions yield different chemotypes with varying proportions of major volatiles (e.g., bornyl acetate, 1,8-cineole, caryophyllene oxide) and sesquiterpene lactones, helping to explain inconsistencies in antibacterial spectra and cytotoxic potencies reported in the literature [60,61,71]. This nuance is crucial when comparing studies and underscores the value of chemotype-aware reporting and standardized bioassays in future work [60,61,71].

A second nuance concerns preparation type. Essential oils and hydrosols can exhibit antimicrobial activity but often differ in potency and target range compared with crude methanolic or aqueous extracts. While essential oils may show pronounced antimicrobial effects, crude extracts frequently yield stronger antioxidant activity and can display notable cytotoxicity in some cancer cell lines, potentially due to the cumulative action of polyphenols, flavonoids, terpenoids, and SLs [48,60,62]. These distinctions highlight the importance of aligning preparation type with the intended bioactivity in both research and application contexts (e.g., food preservation *vs.* therapeutics) [67,72].

A final point of nuance relates to safety and selectivity. While some studies report cytotoxic effects on cancer cells with limited toxicity toward normal cells in certain extracts, others stress the need for comprehensive toxicological assessment given the potent bioactivity of SLs and terpenoids [48]. This balance between efficacy and safety remains to be established *in vivo* for most *D. viscosa* preparations, and careful dose-optimization studies are needed to evaluate therapeutic windows and potential side effects [69,74,77].

Synthesizing across a broad swath of evidence, *D. viscosa* and its chemical constituents demonstrate coherent antioxidant, antibacterial, and antitumor activities, underpinned by phytochemical diversity and influenced by regional chemotypes and extraction approaches [48] (Table 4). The antioxidant capacity is robust and closely tied to polyphenolic and flavonoid contents, particularly in methanolic leaf extracts, with essential oil and hydrosol fractions contributing in an assay-dependent manner [60,62,71,74]. Antibacterial activity is well-supported across extracts and essential oil fractions [60,61,62,69], with activity against common pathogens such as *E. coli*, *S. aureus*, and *Bacillus* species, though the precise spectrum and potency vary with chemotype and assay method; essential oils often provide distinct antibacterial profiles relative to methanolic extracts [67,76,77]. Antitumor activity is notably represented by tomentosin and inuviscolide [48,60,62], with *in vitro* evidence for apoptosis induction and ROS-mediated mechanisms; terpenoid-rich fractions and volatile constituents also show antiproliferative effects in several cancer cell lines, pointing to shared mechanistic themes of apoptosis and oxidative stress across preparations [67,74,81].

Therefore, *D. viscosa* stands as a phytochemically rich and pharmacologically promising plant with demonstrated antioxidant, antimicrobial, and antiproliferative activities that are robust *in vitro* and supported by multiple independent lines of evidence. The field would benefit from harmonized bioscreenings, expanded *in vivo* studies, and a deeper elucidation of structure–activity relationships among tomentosin, inuviscolide, and the broader pool of terpenoids and polyphenols encoded in this species. Such efforts would help to translate these striking *in vitro* observations into clinically or industrially relevant applications, while clarifying safety profiles and therapeutic windows across regional chemotypes.

## 8. Agricultural Applications of *D. viscosa* in Integrated Pest Management and Attraction of Beneficial Insects

*D. viscosa* also occupies a central place in discussions of habitat management within Mediterranean agroecosystems. Rather than being treated solely as a weed, *D. viscosa* is increasingly recognized as a potential component of integrated pest management (IPM) and as a floral resource that can support beneficial arthropods. The evidence compiled from multiple contexts, olive groves, olive- and tomato-based cropping systems, and peri-urban or greenhouse landscapes, points to a nuanced role for *D. viscosa*: it can function as a reservoir and refuge for natural enemies, provide adult nectar and pollen resources, and potentially facilitate predator–prey networks that suppress pests. At the same time, the plant may harbor phytophagous species or pathogens under certain conditions, highlighting the need for context-specific deployment and monitoring within IPM programs. This synthesis integrates findings from diverse studies to discuss mechanisms, crop contexts, potential benefits, risks, and practical recommendations for integrating *D. viscosa* into IPM strategies in the Mediterranean and similar agroecosystems [36,84,85,86,87,88,89,90,91].

### 8.1. D. viscosa as a Habitat and Reservoir for Natural Enemies

Across the Mediterranean, *D. viscosa* has repeatedly been observed to harbor diverse natural enemies, including predatory mites (*Phytoseiidae*), mirid bugs, and parasitoids, thereby contributing to local pest regulation. In olive groves, *D. viscosa* plants border and intersperse crops and host a community of arthropods that includes Miridae, Aphididae, Hymenoptera parasitoids, Formicidae, Araneae, and Aleyrodidae, with dynamics strongly linked to plant phenology and local rust infections [36]. The Phytoseiidae assemblage, often dominated by *Typhloseiella isotricha* on rust-damaged *D. viscosa* leaves, exemplifies how rust–phytoseiid interactions can shape predator availability and prey conditioning cues, potentially supporting predation on pest mites in nearby crops [87]. The broader pattern across regions suggests that *D. viscosa* can function as a non-crop reservoir and habitat for beneficial arthropods, including zoophytophagous mirids and parasitoids, which can underpin conservation biological control in olive groves, tomatoes, and other crops when embedded within diversified landscapes [84,85,86,87]. However, the net pest-control effect is context-dependent, mediated by pest–enemy community composition, seasonal phenology, and landscape structure; thus, *D. viscosa*’s contribution to pest suppression is not universal and must be evaluated locally [84,85,86,87].

### 8.2. D. viscosa as a Source of Floral Resources and Convergence with Conservation Biological Control

Flowers in *D. viscosa* provide nectar and pollen that adult natural enemies rely on, especially during periods when crop floral resources are scarce. In olive groves and other Mediterranean systems, non-crop flowering plants, including *D. viscosa*, have been shown to sustain predators and parasitoids by offering nectar/pollen and alternative resources, thereby supporting pest suppression in IPM programs [85,88,89]. Flowering phenology is particularly relevant: *D. viscosa* often blooms in late summer and autumn, a period when crops may have reduced floral resources, which may align with parasitoid and predator activity and support year-round pest management in perennial systems such as olive groves [36,88]. Nevertheless, a limitation is that non-crop flowering plants can also harbor pests or pathogens, so the introduction or preservation of *D. viscosa* as a nectar source should be paired with careful monitoring and integrated into broader habitat management to maximize beneficial outcomes [88,89].

### 8.3. D. viscosa as a Refuge Plant and Facilitator of Predator–Prey Networks for Key Pests

In crop-specific contexts such as tomato and olive systems, *D. viscosa* has been reported to act as a refuge plant that supports generalist predators (notably mirids) and zoophytophagous predators, which can move into crops and contribute to pest suppression. In tomato-centered IPM programs, companion plants similar to *D. viscosa* can bolster predator establishment and movement into crops, complementing releases of biological control agents against pests such as *Tuta absoluta*, a major tomato pest [85,91,92,93]. In olive groves, nectar resources from *D. viscosa* flowers can sustain adult parasitoids and predators, reinforcing IPM in perennial Mediterranean systems [36,85,88]. The caveat remains that *D. viscosa* may attract or harbor pests under certain conditions, necessitating careful site selection and monitoring to ensure that the habitat benefits outweigh any potential pest risks. This nuanced view aligns with broader IPM literature recognizing trade-offs in habitat manipulation and the need for multilayered monitoring and adaptive management to optimize outcomes [88,89,94].

### 8.4. D. viscosa in the Context of Tuta Absoluta and Other Pests

The *Tuta absoluta* literature emphasizes habitat management and indigenous natural enemies as core components of IPM, particularly in greenhouse and field tomato systems. *D. viscosa* is frequently cited as a refuge plant that can support natural enemies and promote pest suppression in tomato crops within the Mediterranean context; however, direct, crop-specific experimental trials directly isolating the effect of *D. viscosa* on *T. absoluta* performance are limited in the cited materials. The broader implication is that incorporating *D. viscosa* within a habitat suite, alongside banker plants, companion flora, and native natural enemies, can bolster IPM by sustaining predator and parasitoid populations, especially in Mediterranean cropping systems where *D. viscosa* is prevalent [36,84,85,91,93]. IPPM framings further encourage the integration of habitat management with pollinator protection and monitoring, recognizing that the same flora that supports natural enemies may also influence pollinators and non-target insects [89,90].

### 8.5. Practical Considerations for Implementation

Landing *D. viscosa* within an IPM program requires careful landscape design and ongoing monitoring. Landscape planning should position *D. viscosa* as part of a semi-natural habitat around fields and nurseries, ensuring its floral resources are available during periods when parasitoids and predators are active in crops. Plant phenology should be synchronized with crop phenology to maximize nectar/pollen availability during times of predator and parasitoid activity, particularly late summer and autumn when crop resources may be scarce [36,88,89]. In tomato and olive systems, *D. viscosa* can function as a companion or banker plant to support *Miridae* and *Phytoseiidae* populations, respectively, but requires monitoring to evaluate net pest suppression and to detect any unintended pest proliferation or virus reservoirs [85,92,94]. Monitoring should be integrated with disease surveillance and pest scouting to quickly detect any adverse associations and adapt habitat management accordingly [87,88,94]. Finally, *D. viscosa* habitat management should be integrated with other IPM tools, including semiochemicals, banker plants, and selective biocontrol releases, to realize synergistic effects and reduce reliance on pesticides while safeguarding pollinators and beneficial insects—embodying IPPM principles in practice [89,93,95].

### 8.6. Nuances and Points of Disagreement

The bulk of evidence supports a beneficial role for *D. viscosa* as a habitat/resource for natural enemies in several Mediterranean contexts, but there is no universal outcome. The degree of pest suppression observed is context-dependent: some studies document abundant *Phytoseiidae* and parasitoids on *D. viscosa*, while others emphasize the plant’s potential to harbor phytophagous pests or pathogens under particular conditions [36]. Consequently, site-specific risk assessment and adaptive management are essential when deploying *D. viscosa* as part of an IPM strategy. The literature consistently advocates integrating habitat management with broader IPM tools to maximize ecosystem services and to avoid unintended consequences, a stance aligned with IPPM frameworks that aim to balance pest suppression with pollinator and biodiversity considerations [87,88,89].

Therefore, *D. viscosa* has potential as a multi-functional component of Mediterranean IPM by serving as a non-crop reservoir and habitat for natural enemies, providing nectar/pollen resources for adult beneficials, and functioning as a refuge plant that supports predator–prey networks in crops such as olives and tomatoes. Realizing these benefits requires landscape-level planning, phenology-aware deployment, and robust monitoring to detect any pest amplification or pathogen issues [84,85,86]. *D. viscosa* should be incorporated within diversified habitat-management designs that align with IPPM principles, combining it with banker plants, semiochemicals, and selective biocontrol releases to maximize pest suppression, pollinator protection, and overall biodiversity benefits. Where pest pressures are high or disease risks are present, adaptively managing the habitat mosaic and continually integrating new evidence will be essential for achieving net beneficial outcomes [88,89,90,91,93].

## 9. *Dittrichia viscosa* in the Phytoremediation of Degraded Soils: A Discursive Synthesis of Sustainability Implications

*D. viscosa* has also attracted attention as a candidate species for phytoremediation in degraded soils, particularly in mining-affected and semiarid Mediterranean landscapes. The sustainability of using *D. viscosa* for soil remediation hinges on its capacity to stabilize contaminants in situ, its biomass production, and its interactions with soil biota, which together frame a Gentle Remediation Options (GROs) approach rather than a sole emphasis on phytoextraction [40,41]. Across the available evidence, *D. viscosa* consistently exhibits high biomass, robust tolerance to metal stress, and a tendency to sequester metals primarily in roots and below-ground tissues, patterns that underpin its potential role in phytostabilization and broader phytomanagement strategies rather than rapid decontamination through extraction alone [96,97]. This discourse integrates the core themes from multiple sources, emphasizing both consensus and nuance, and foregrounds implications for research design and land management.

## 10. Biomass Traits, Valorization, and Their Role in Sustainable Circular Systems

The ecological profile of *D. viscosa*, its status as a pioneer shrub with vigorous biomass and a broad Mediterranean distribution, underpins its practicality for in situ stabilization. The literature characterizes *D. viscosa* as a metallophyte capable of thriving in soils with and without heavy-metal contamination, a trait that supports establishment on degraded lands where other vegetation might struggle. Importantly, while its bioaccumulation in leaves and roots confirms its ability to harbor trace elements, the reported bioaccumulation factor (BAF) and translocation factor (TF) values generally do not reach the thresholds required for effective phytoextraction in highly contaminated sites; instead, the high biomass combined with root- and litter-associated metal pools makes it well-suited for phytostabilization and GROs. This nuanced view—high biomass and tolerance, but limited phytoextraction efficiency—frames *D. viscosa* as a stabilization-oriented component of a remediation portfolio rather than a stand-alone phytoextractor [40].

In practice, this aligns with the GRO framework, which prioritizes in situ stabilization, erosion control, and long-term containment over radical ex situ decontamination when contaminated sites are extensive or when remediation costs and ecological disruption would be prohibitive. Several sources corroborate the view that *D. viscosa* can function effectively within photostabilization and GRO strategies, contributing to the stabilization of contaminants in the root zone and reducing pollutant mobility and dust dispersion, thereby mitigating ecological risk while allowing for continued land use and biomass production [40,96,97]. The emphasis on stabilization is not a concession to lower remediation aspirations but a recognition of the practical dynamics of field-contaminated environments and the need to balance ecological integrity with feasible management actions [40,97].

A compelling sustainability argument for using *D. viscosa* lies in its substantial biomass, which opens pathways for biomass valorization, most plausibly in energy or material streams compatible with a phytomanagement approach. In the Mediterranean setting, phytostabilization coupled with biomass use—such as bioenergy production—has been explored as a route to pair remediation with renewable energy outcomes, provided contaminants do not preclude safe energy recovery and ash disposal. The relevant literature documents several bioenergy pathways for TE-contaminated plant biomass, noting that energy yields and combustion properties must be weighed against contaminant loads and regulatory constraints. Notably, biomass from *D. viscosa* and related metallophytes has been considered suitable for energy-focused end uses within phytostabilization programs, contributing to a net environmental gain when integrated into sustainable land management and energy portfolios under Mediterranean climatic conditions [96,98].

Yet, this avenue requires site-specific assessment of contaminant loads, HHV (higher heating value), and the fate of contaminants in ash, as well as life-cycle environmental impacts. The potential to couple remediation with biomass-derived energy supports sustainability by turning remediation waste into value-added outputs, aligning with circular economy principles and land reclamation goals. However, regulatory constraints and risk management considerations regarding contaminant concentrations in harvested biomass must be carefully evaluated to avoid secondary environmental or health risks [96,98].

### Site Specificity and the Limits of Phytoextraction

A key nuance across the evidence base is that *D. viscosa* is not a hyperaccumulator with uniformly high phytoextraction potential. In multiple field-scale assessments, TF and BAF metrics indicate limited capacity for rapid or complete removal of contaminants under high contamination. Consequently, the dominant sustainable role for *D. viscosa* is within GROs that prioritize stabilization and ecological restoration, particularly in degraded Mediterranean soils where erosion control, soil structure improvement, and habitat restoration are pressing objectives. This interpretation is supported by comparative studies and reviews that position *D. viscosa* as a robust stabilizer with significant biomass, often used in combination with soil amendments, AMF inoculation, and rhizoremediation strategies to enhance containment and soil health outcomes [40,41,97].

There is ongoing scholarly debate about the precise role of AMF and endophytic communities in modulating metal uptake for *D. viscosa*, with some studies indicating that AMF can inhibit certain metal translocations while others emphasize their overall positive contribution to stabilization. The variance underscores the importance of site-specific experiments and long-term monitoring when deploying *D. viscosa* for sustainable remediation. In practice, this means that practitioners should avoid over-relying on *D. viscosa* for phytoextraction in highly contaminated sites and instead plan for a portfolio of GRO strategies, where *D. viscosa* contributes to stabilization, habitat restoration, and potential biomass valorization within a carefully designed, monitored framework [40,41,99].

## 11. Mechanistic Underpinnings: Rhizosphere, Mycorrhiza, and Endophyte Interactions

A mechanistic reading of sustainability benefits highlights the role of rhizosphere processes and plant–microbe interactions in modulating metal bioavailability and plant tolerance. Arbuscular mycorrhizal fungi (AMF) and dark septate endophytes (DSE) are repeatedly implicated as important modulators of metal uptake, translocation, and stress tolerance in metallophytes, including *D. viscosa*. AMF associations often reduce shoot metal accumulation while enhancing root structural and physiological resilience, thereby promoting phytostabilization. Endophytic associations similarly influence metal tolerance and overall plant health, potentially altering the balance between stabilization and translocation. While the magnitude and direction of AMF effects can be site-specific, the consensus across studies is that these biotic interactions are central to the sustainability advantages of using metallophytes in polluted landscapes: they bolster plant establishment, improve nutrient and water uptake under stress, and shape metal partitioning in ways that support in situ containment rather than unabated extraction [40,41,99,100].

From a management perspective, incorporating AMF inoculation or fostering native mycorrhizal networks could be a practical lever to maximize stabilization outcomes and restore soil health. However, the literature also notes that AMF effects on metal uptake can vary with soil pH, contaminant speciation, and host species genotype, underscoring the need for context-specific design and monitoring. This nuance is essential for sustainability assessments, because the same inoculation strategy might yield different stabilization outcomes depending on local edaphic and pollution conditions [40,41,99].

## 12. Practical Implications for Research and Land Management

Beyond theory, actionable guidance emerges from field-oriented recommendations. Field trials should be designed to quantify stabilization efficacy by measuring changes in soil metal mobility, leachate concentrations, and plant tissue distribution over multiple years, thereby enabling a robust assessment of containment performance and the potential risks of shoot translocation under varying contamination regimes. Simultaneously, integrating AMF inocula and monitoring colonization rates can optimize stabilization outcomes, while keeping an eye on the trade-offs between root- and shoot-associated metal partitioning and harvestable biomass yields. Investigations into rhizosphere management, including bioaugmentation or designer rhizospheres, offer avenues to further support soil health restoration and stabilization in polluted substrates [40,41,99].

From an economic and policy perspective, exploring biomass-to-energy pathways as part of a broader phytomanagement strategy can contribute to the sustainability portfolio, especially if energy yields are high and contaminant handling is compliant with regulations. Life-cycle analyses and site-specific risk assessments are essential to ensure that biomass valorization does not introduce new environmental or health burdens. This approach dovetails with the broader discourse on AML (abandoned mine lands) reclamation, where plant-based remediation technologies are favored not only for their ecological compatibility but also for public acceptance and potential co-benefits in ecosystem services, biodiversity support, and climate regulation through soil carbon dynamics [96,97,98].

### Nuances and Areas for Further Research

A persistent nuance across sources is the context-dependence of AMF and endophyte effects on metal uptake in *D. viscosa*, suggesting that generalizations should be avoided. Site-specific experiments are needed to quantify how mycorrhizal associations modulate metal immobilization in the rhizosphere and root zones, and to determine whether inoculation strategies measurably improve stabilization without compromising biomass yields or ecosystem functions. Longitudinal field studies are also needed to verify the stability of immobilized contaminants over time and across seasonal cycles, particularly in semiarid climates where erosion and hydrological fluxes can influence contaminant mobility. Finally, while biomass valorization holds promise, regulatory and safety considerations surrounding contaminated biomass—especially for energy conversion and ash disposal—must be integrated into sustainability assessments to prevent unintended environmental trade-offs [40,41,96,97,99].

## 13. Conclusions and Perspectives

*D. viscosa* emerges from the current literature as a highly versatile Mediterranean species in which volatile organic compounds (VOCs) play a central and integrative role across chemical, ecological, and functional dimensions. The evidence reviewed here highlights that its volatile fractions, primarily composed of mono- and sesquiterpenes, are not only chemically diverse and strongly influenced by geographic and environmental factors, but also deeply involved in mediating biological activities and ecological interactions.

A key insight of this review is that VOCs should not be considered isolated metabolites but rather as part of a broader, dynamic phytochemical system in which volatile and non-volatile compounds act in concert. This integrated perspective is particularly relevant for *D. viscosa*, where chemotypic variability, glandular trichome activity, and environmental modulation collectively shape a complex and context-dependent metabolite profile. In this framework, VOCs represent both functional drivers of ecological success, through plant–insect and plant–microbe interactions, and promising bioactive agents with applications in agriculture, pharmacology, and biotechnology.

However, the transition of *D. viscosa* from an empirically recognized botanical resource into a reliably exploited biotechnological agent is currently bottlenecked by five major research gaps:

- Protocol standardization: A lack of harmonized extraction, distillation, and analytical methodologies prevents the reliable cross-study comparison of VOC yields and chemical profiles.
- Chemotype classification: The pronounced geographic, seasonal, and organ-specific variability of the species requires a formalized, standardized system for mapping Mediterranean chemotypes.
- Compound activity validation: While bioactivity is widely reported for crude extracts and whole oils, targeted structure–activity relationship (SAR) studies are required to experimentally validate the specific molecular targets of individual constituents.
- Comprehensive toxicological profiling: Despite promising *in vitro* selectivity, systematic *in vivo* toxicity and dose-optimization studies remain necessary to establish safe therapeutic windows for sesquiterpene lactones and terpenoid fractions.
- Field-scale ecological testing: The semiochemical, allelopathic, and tri-trophic functions of VOC emissions must be transitioned from controlled laboratory headspaces to realistic, open-field agroecological trials.

In conclusion, *D. viscosa* is a compelling model species in which VOCs serve as a unifying element linking phytochemistry, ecology, and bioactivity. Fully understanding and harnessing this volatile dimension will be essential for translating the plant’s multifunctional potential into practical applications, making VOCs not only a defining feature of *D. viscosa*, but also a key resource for future research and innovation.

## Figures and Tables

**Table 1:** Summary of the main taxonomic, morphological, anatomical, ecological, physiological, and functional traits of *Dittrichia viscosa* reported in the literature. The table highlights the species’ diagnostic characteristics, habitat preferences, stress adaptation, ecological interactions, phytoremediation potential, seed germination responses to salinity, and currently available genomic resources.

Trait Category	Specific Trait or Note	Bibliography
Taxonomic status	Currently accepted basionym (syn. *Inula viscosa*). Definitive classification requires an integrative approach (molecular phylogenetics + anatomy).	[16,32,33]
Growth form and habitat	Perennial shrub/herb widespread across the Mediterranean. Opportunistic pioneer species favoring disturbed soils, roadsides, and coasts.	[16,35,36]
Micromorphology of achenes	Oblong-elliptic shape with a rugulose surface. Surface ornamentation serves as a diagnostic marker against close relatives (e.g., *D. graveolens*).	[34]
Pollen ultrastructure	Exhibits a Senecioid exine pattern with a 3-layer tectal complex. Serves as a supportive, rather than standalone, taxonomic trait.	[32]
Leaf and stem anatomy/micromorphology	Provides secondary diagnostic features for the tribe Inuleae. Must be interpreted alongside molecular data.	[16,32,33]
Drought and salinity adaptation	Highly tolerant to summer drought. Moderately salt-tolerant; occupies salt-marsh margins via niche partitioning.	[35,39]
Biomass production and persistence	Produces rapid, high-density vegetative cover. Highly persistent in semi-arid and degraded substrates.	[16,35,36]
Soil/microbial associations	Forms symbiotic networks with AMF and dark septate endophytes (DSE). Microbes regulate metal uptake and boost host stress tolerance.	[39,41,42,44]
Phytoremediation potential	Functions as a phytostabilizer (Gentle Remediation Options) rather than a hyperaccumulator. Containment occurs primarily via root-zone immobilization.	[39,40,42,43,44]
Arthropod interactions and ecosystem services	Acts as a natural non-crop reservoir for beneficial predators and parasitoids. Supports conservation biological control in olive/cropping systems.	[36,44]
Invasiveness outside native range	Documented invasive species in Australia and North America. Displays high potential for climate-change-driven range expansion.	[15,16,35,42]
Seed germination responses to salinity	Maintains viable seed germination under moderate salt stress. Germination rate correlates inversely with high salinity concentrations.	[57,58]
Genome resources (*D. graveolens*)	Chromosome-level assembly of sister species *D. graveolens* serves as a comparative proxy. Enables functional studies on range expansion and climatic adaptation.	[59]

**Table 2:** Comparative overview of volatile organic compounds (VOCs) reported for *Dittrichia viscosa* across different geographical regions. The table summarizes the plant organ analyzed, sample type (essential oil, hydrosol, or volatile fraction), extraction or sampling method, analytical technique, predominant volatile constituents, reported biological activities, and corresponding literature references, highlighting the pronounced chemotypic variability of the species.

Country/Region	Plant Part	Sample Type	Extraction/Sampling	Analytical Method	Major VOCs	Main Reported Biological Activity	Reference
Croatia	Aerial parts	Essential oil	Hydrodistillation	GC–MS	Caryophyllene oxide, 1,8-cineole, α-muurolol	Antimicrobial, antiproliferative, antiphytoviral	[60]
Croatia	Aerial parts	Hydrosol	Hydrodistillation	GC–MS	p-Menth-1-en-9-ol, cis-sabinene hydrate, linalool	Antimicrobial, antiphytoviral	[60]
Algeria	Aerial parts	Essential oil	Hydrodistillation	GC–MS	Polygodial, phytol, intermedeol, fokienol	Antibacterial	[61]
Morocco	Leaves	Essential oil	Hydrodistillation	GC–MS	Caryophyllene oxide, borneol, bornyl acetate	Antioxidant, antimicrobial, insecticidal	[62]
Algeria	Roots	Essential oil	Hydrodistillation	GC–MS	β-Acorenol and related terpenoids	Antioxidant, antimicrobial, cytotoxic	[66]
Algeria	Roots	Hydromethanolic extract	Solvent extraction	UHPLC/chromatographic profiling	Polyphenols (hesperetin, naringenin, kaempferide, tangeretin)	Antioxidant, cytotoxic	[66]
Morocco	Leaves	Essential oil	Hydrodistillation	GC–MS	Chemotype dominated by oxygenated terpenes	Antioxidant, antimicrobial, antidiabetic (*in vitro*; molecular docking)	[67]
Mediterranean populations	Leaves/aerial parts	VOCs (review)	Different extraction methods	GC–MS, GC–FID/MS, HS-SPME/GC–MS	1,8-Cineole, caryophyllene oxide, borneol, bornyl acetate, isocostic acid	Comparative chemotype analysis; geographical variability	[46]
Mediterranean populations	Leaves/aerial parts	VOCs	Various	Various	Terpenoid-rich volatile profiles	Phytotoxic and allelopathic potential	[64]

**Table 3:** Summary of the chemical constituents reported in *Dittrichia viscosa* across different plant organs and geographical regions. The chemical profile is organized into four sections: (**A**) volatile organic compounds (VOCs), (**B**) essential oil constituents, (**C**) hydrosol constituents, and (**D**) non-volatile phytochemicals. Each section classifies the identified compounds according to their chemical family and reports the corresponding plant source, geographical context, and representative biological or ecological relevance.

Chemical	Chemical Class/Subclass (with Subcategory)	Plant Part(s) and Regional Context	Representative Roles/Context	References
(**A**) Volatile organic compounds (VOCs).				
α-Pinene	Terpene (monoterpene)	Headspace volatiles across samples	Representative headspace component; site-specific variation	[62] general headspace data across populations.
β-Myrcene	Terpene (monoterpene)	Headspace volatiles	Common headspace constituent; variable by site	[62] headspace reports.
p-Cymene	Terpene (monoterpene)	Headspace volatiles	Part of volatile bouquet; population-dependent	[62] headspace analyses.
D-Limonene	Terpene (monoterpene)	Headspace volatiles	Frequent headspace constituent; ecological relevance	[62] headspace analyses.
(**B**) Essential oil constituents.				
Polygodial	Terpene (sesquiterpene dialdehyde; lactone)	Algerian *Inula viscosa* EO; chemotypes	Major EO constituent in several Algerian populations; antimicrobial context	[61] describe Algerian chemotypes and major components in *Inula viscosa* EO; broader context of terpene-rich EO in *D. viscosa* is supported by multiple North African studies.
Phytol	Terpene (diterpenoid; acyclic diterpene alcohol)	EO of Algerian *Inula viscosa*	Major EO constituent in Algerian chemotypes; contributes to terpene-rich EO profile	[61] report phytol as a major EO constituent in Algerian populations.
Intermedeol	Terpene (sesquiterpene)	EO of Algerian *Inula viscosa*	Major EO component in some Algerian chemotypes	[61] note intermedeol among Algerian EO constituents.
Caryophyllene oxide	Terpene (sesquiterpene oxide)	EO of Algerian *Inula viscosa*; other populations	Noted major EO constituent; variable across populations	[61] document caryophyllene oxide presence in Algerian EOs.
Nerolidol (E/Z)	Terpene (sesquiterpene alcohol)	Algerian EO analyses; broader studies	Significant EO constituent; chemotype-dependent abundance	[61] report nerolidol as a variable major component in Algerian chemotypes.
β-Caryophyllene	Terpene (sesquiterpene)	EO profiles across *D. viscosa* (Algeria and elsewhere)	Common EO component; regional variability	[61] describe β-caryophyllene presence and variability.
Bornyl acetate	Terpene (monoterpene ester)	Algerian EO chemotypes	Major component in certain chemotypes	[61] report major components including bornyl acetate in Algerian EO.
Bornol (borneol)	Terpene (monoterpene alcohol)	EO fractions	Noted monoterpene in EO compositions	[61] other Algerian EO reports mention borneol as a component.
α-Terpinyl acetate	Terpene (monoterpene ester)	Algerian EO constituents	Reported among Algerian EO constituents	[61] note monoterpene esters in Algerian chemotypes.
Camphor-related derivatives	Terpene (monoterpene relatives)	EO of some populations	Dominant monoterpenes in certain chemotypes	[60,61] document camphor-type constituents in Algerian EO.
Fokienol	Terpene (sesquiterpene)	EO of Algerian chemotypes	Major component in some Algerian chemotypes	[61] related Algerian EO profiles.
Costic acid	Terpene (sesquiterpene lactone/acid)	EO/terpenoid fractions (roots/leaves)	Organ-specific bioactivity; context-dependent	[66] root/EO contexts document Costic acid–related chemistry.
(**C**) Hydrosol constituents.				
1,8-Cineole (eucalyptol)	Terpene (monoterpene oxide)	Hydrosol; headspace volatiles across samples	Major hydrosol volatile; polar fraction; antimicrobial context	[60] Headspace/WS analyses support 1,8-cineole as a key hydrosol volatile.
3,4-Dihydroxybenzoic acid	Phenolic acid	Hydrosol	Hydrosol antimicrobial context; polar volatile complement	[60]
1,8-Cineole; Linalool	Terpenes (monoterpenes; oxygenated)	Hydrosol volatiles; headspace	Hydrosol polar volatiles with antimicrobial activity	[62]
3,4-Dihydroxybenzoic acid (repeat)	Phenolic acid	Hydrosol	Hydrosol antimicrobial context	[61]
(**D**) Non-volatile phytochemicals.				
Lupeol	Terpene (triterpene)	Terpenoid-rich fractions (leaves/stems)	Found in terpenoid-rich fractions; anticancer context	[72] terpenoid-rich fractions reporting lupeol.
Isopulegol	Terpene (monoterpene)	Terpenoid-rich fractions	Enriched in terpenoid fractions; bioactivity notes	[72] report isopulegol in terpenoid fractions.
Betulin	Terpene (triterpene)	Terpenoid-rich fractions	Detected in terpenoid-rich fractions; bioactivity context	[72]
Quercetin	Flavonoid (flavonol)	Leaves; multiple extracts	Central antioxidant/enzyme-inhibitory flavonoid	[49,66,70] document quercetin presence in leaves and extracts.
Quercetin glycosides (e.g., isoquercitrin)	Flavonoid glycoside	Leaves; UHPLC profiling	Glycosylated forms common; antioxidant context	[49,70,72] report isoquercitrin among leaf phenolics.
Kaempferol	Flavonoid (flavonol aglycone)	Leaves; extracts	Common flavonol in *Dittrichia viscosa* leaves	[49,70,72]
Kaempferol glycosides	Flavonoid glycosides	Leaves	Glycosides detected in leaf profiles	[49,70]
Luteolin	Flavonoid (flavone)	Leaves; extracts	Detected in flavonoid panels; bioactivity context	[49,70]
Apigenin	Flavonoid (flavone)	Leaves; extracts	Detected in leaves; glycosides reported	[49,70]
Quercetin-3-O-glucoside (isoquercitrin)	Flavonoid glycoside	Leaves	Common quercetin glycoside; antioxidant context	[49,70]
Rutin (quercetin-3-O-rutinoside)	Flavonoid glycoside	Leaves	Important flavonoid glycoside in *D. viscosa* leaves	[49,70]
Catechin	Flavonoid (flavanol)	Leaves; leaf extracts	Detected in leaf extracts; antioxidant context	[49,70]
Catechin gallate	Flavonoid (flavanol gallate)	Leaves	Reported as gallate derivative in inventories	[49,70]
Epicatechin	Flavonoid (flavanol)	Leaves	Reported in polyphenol scans	[49,70]
Taxifolin	Flavonoid (dihydroflavonol)	Leaves	Noted in polyphenol datasets; DHF class	[66,72]
Chlorogenic acids; dicaffeoylquinic acids	Phenolic acids	Leaf EtOAc/hydroethanolic extracts; Algerian/North African material	Major phenolics; antioxidant activity context	[49,66,70]
Rosmarinic acid	Phenolic acid	Leaf extracts	Common in leaves; antioxidant capacity	[49,66,70]
Caffeic acid derivatives (dicaffeoylquinic acids, etc.)	Phenolic acids	North African leaf extracts	Major phenolic family; bioactivities	[49,66,70]
1,5-O-Caffeoylquinic acid	Phenolic acid	Algerian/North African leaves	Reported as major phenolic in ethanolic extracts	[66,70]
Dihydrobenzofuran lignans (cinchonain-type)	Phenolics/lignans	Leaves; nutraceutical profiling	Unusual lignan derivatives; antioxidant/cytotoxic context	[66,72]
Shikimoyl depsides of caffeic acid	Phenolics	Leaves; nutraceutical profiling	Unique phenolic subclasses; bioactivity context	[66,72]
Costic acid	Terpene (sesquiterpene lactone/acid)	Root and leaf terpenoid fractions	Organ-specific bioactivity; context-dependent	[66,72]
Lupeol	Terpene (triterpene)	Terpenoid-rich leaf/stem fractions	Anticancer context in terpenoid-rich fractions	[72]
Hesperetin; Naringenin	Flavonoids (flavanones)	Root extracts; some leaf profiles	Occurrence in polyphenol surveys; bioactivities noted	[66,72]

The table integrates compounds across essential oil analyses, hydrosol studies, and non-volatile leaf/root extracts. Groupings reflect the main literature classifications: terpenes (including monoterpenes, sesquiterpenes, and terpenoid derivatives), flavonoids (aglycones and glycosides), and hydroxycinnamic acids/other phenolics (including lignans and depsides where reported). Where the literature clearly distinguishes glycosides *vs.* aglycones, we have annotated subcategories accordingly (e.g., isoquercitrin *vs.* quercetin; rutin *vs.* quercetin). Where sources report a compound only at the class level, we have indicated the context and noted any ambiguity. Cross-study concordance varies with geography, organ, and extraction method, which is consistent with the chemotype concept for *D. viscosa*. When applicable, hydrosol constituents are distinguished from EO components to reflect their different polarities and bioactive profiles, as reported in the hydrosol-focused literature [60], complementary headspace and EO analyses [55], and organ-specific terpenoid inventories [48,66,72].

**Table 4:** Summary of the biological activities reported for *Dittrichia viscosa* and its major phytochemicals. The table highlights the antioxidant, antibacterial, and antitumor properties described in the literature, together with the influence of chemotype, geographical origin, plant material, and extraction method on the reported biological activities.

Entity	Antioxidant Activity	Antibacterial Activity	Antitumor Activity	Notes on Chemotype/Region or Preparation	Other Biological Activities
*D. viscosa* leaves (methanolic extract)	High phenolic and flavonoid content; strong DPPH and ABTS radical-scavenging; antiglycation effects; frequently higher activity than aqueous extracts; key polyphenols include caffeoylquinic acid derivatives such as 3,4-dicaffeoylquinic acid [48,72].	Not primary; antimicrobial activity reported for extracts/oils in related matrices; synergy with antibiotics noted in leaves-containing matrices [74,79,80]; overall antimicrobial potential supported in methanolic extracts [60,77].	Not primary; but extract cytotoxicity reported against certain cancer cell lines in related studies; *in vitro* antiproliferative effects linked to SLs in extracts [69,74,77].	Methanolic leaf extracts consistently show strongest antioxidant profiles; regional variation affects polyphenol profiles and activity [48,72].	Antiglycation; α-amylase and α-glucosidase inhibition; anti-inflammatory activity reported for phenolic-rich extracts [46,48]
*D. viscosa* essential oil (DVEO)	Antioxidant activity: significant but assay-dependent; borneol/1,8-cineole and other terpenoids contribute to radical scavenging in some assays; oil often slightly less potent than crude methanolic extracts in some tests [60,62,67].	Antibacterial activity: notable activity against several pathogens; composition-driven variability explains differing spectra across studies; major terpenes (1,8-cineole, caryophyllene oxide) implicated in antimicrobial action [60,62,67].	Antiproliferative effects observed for DVEO against HeLa, HCT116, U2OS cell lines in some reports [60].	Chemotype-dependent; Algerian/Turkish Mediterranean populations exhibit variable oil compositions that affect bioactivity [61,71,78].	Anti-inflammatory effects; α-amylase and α-glucosidase inhibition; antidiabetic potential supported by *in vitro* assays and molecular docking [60,67]
*D. viscosa* hydrosol	Antioxidant contributions from hydrosol constituents, including detected phenolic acids (e.g., 3,4-dihydroxybenzoic acid) and volatile terpenoids, may modestly contribute to antioxidant activity; activity is assay-dependent [60,74].	Antibacterial effects present; hydrosol shows antimicrobial activity; composition-dependent [60].	Antiproliferative effects observed for hydrosol-treated cancer cell lines; suggested involvement of oxidative stress (GSH perturbation) in tumor cells [60].	Hydrosol composition overlaps with that of essential oil; differences in fractions contribute to variability in activity [60].	No additional biological activities clearly demonstrated.
Tomentosin (sesquiterpene lactone; widely present in *D. viscosa*)	Not a primary antioxidant; SLs are largely linked to antiproliferative effects; some ROS-related context in tumor models, but antioxidant claims are mainly polyphenol-driven [74,81].	Not primary; antimicrobial contributions mainly from essential oil/whole extracts; SLs show antimicrobial effects in related *Inula* species; specific antibacterial data for tomentosin-rich fractions are limited in this set [74,81].	Strong antiproliferative activity across multiple cancer cell lines; mechanisms include apoptosis induction, ROS generation, cell-cycle arrest, covalent modification of cysteine residues and proteasome-related pathways cited [60,74,81].	Key SLs (tomentosin) highlighted for anticancer potential; regional extraction can yield tomentosin-rich fractions (ethanol/ethyl acetate extracts) with pronounced cytotoxicity [74,77].	Anti-inflammatory activity; apoptosis induction; proteasome inhibition [46]
Inuviscolide (sesquiterpene lactone)	Not a primary antioxidant; context mainly on antiproliferative activity via SLs [74,81].	Limited direct antibacterial data; antimicrobial activity is more closely linked to oil/extract matrices containing SLs and other constituents [62,67].	Noted contributor to antiproliferative activity in *D. viscosa* extracts; works in concert with tomentosin in some fractions to induce apoptosis in cancer cells [60,74,81].	Often studied together with tomentosin in ethyl acetate/ethyl acetate-rich extracts showing strong cytotoxicity [74,77].	Anti-inflammatory and pro-apoptotic activities [46]
*D. viscosa* ethanolic extracts (Morocco/Algeria samples)	Phenolic profiling by UHPLC-DAD-ESI/MS identifies prominent phenolics (17 compounds; e.g., 1,5-O-caffeoylquinic acid); cytotoxic activity against AGS and A549 lines; ethanol extracts show notable antioxidant content and activity in DPPH/ABTS assays [70,72].	Antibacterial activity reported for ethanol extracts in various contexts; essential oils more prominent in oil-based matrices, but ethanol extracts contribute to antimicrobial profiles in some reports [70,72].	Cytotoxic effects against AGS and A549 cell lines; death pathways involve caspase-independent mechanisms and RIPK1/necroptosis; notable selectivity in some contexts [70].	Regional Moroccan/Algerian origin; solvent choice (ethanol) influences phytochemical profile and bioactivity; notable upregulation of death pathways in cancer cells [70].	α-Amylase and α-glucosidase inhibition; anti-inflammatory effects; potential antidiabetic activity [46,48,73]
*D. viscosa* leaf water extract/aqueous fractions	Moderate antioxidant activity; DPPH/ABTS results vary with extraction; some reports show high phenolic content in aqueous fractions [60,82].	Antibacterial activity reported in some aqueous extracts; chemospectrum influenced by solvent and plant part [60].	Antitumor effects reported in some extracts *in vivo* and *in vitro* (e.g., colon cancer models in mice and cell lines) with varying mechanisms including apoptosis and proteasome inhibition; significant evidence from skin cancer and colon cancer models *in vivo* and *in vitro* [60,73].	Extraction solvent and plant part influence content and bioactivity; offers potential for chemopreventive uses and combination strategies [60,73].	No additional biological activities clearly demonstrated.
*Dittrichia graveolens*/related Inula species (contextual comparator)	Antioxidant and antitumor activities linked to terpenes and flavonoids including tomentosin/inuviscolide; serves as a comparative reference for SL-rich *Inula* spp. [76,77,82].	Antimicrobial and antiproliferative activities documented; chemotypes influence spectra; serves to contextualize *D. viscosa* findings [76,77].	Anticancer activity reported with SLs in Inula/Dittrichia taxa; synthesis of mechanistic themes across species [74,77,82].	Highlights the broader Inuleae-Inulinae metabolite landscape; emphasizes chemotype-dependent variability [83].	

## Data Availability

No new data were created or analyzed in this study. Data sharing is not applicable to this article.
